# XueFu ZhuYu Decoction Alleviates Cardiopulmonary Bypass-Induced NLRP3 Inflammasome-Dependent Pyroptosis by Inhibiting IkB-*α*/NF-*κ*B Pathway in Acute Lung Injury Rats

**DOI:** 10.1155/2022/6248870

**Published:** 2022-09-10

**Authors:** Hui Li, Wenlei Zhang, Qiaoqin Lou, Yuejin Chang, Zhenhao Lin, Lingli Lou

**Affiliations:** Department of Intensive Care Unit, Hangzhou Third People's Hospital, Zhejiang, Hangzhou, China

## Abstract

XueFu ZhuYu Decoction (XFZYD) is an effective prescription that is widely used to improve blood circulation by removing blood stasis. This study aimed to investigate the effects and the underlying molecular mechanisms of XFZYD on lung pyroptosis in cardiopulmonary bypass- (CPB-) induced acute lung injury (ALI) rats. A rat model of ALI was induced by CPB treatment after XFZYD, Ac-YVAD-CMK, and Bay-11-7082 administration. The respiratory index (RI) and oxygenation index (OI) were determined at each time point. The levels of interleukin (IL)-1*β*, IL-6, IL-18, and TNF-*α* in serum and lung were measured by enzyme-linked immunosorbent assays (ELISA). Moreover, the protein levels, neutrophil counts, and total cell of bronchoalveolar lavage fluid (BALF) were detected. Additionally, Myeloperoxidase (MPO) expression was detected by immunohistochemical assay. Lung injury was evaluated with the wet/dry (W/D) ratio and pathologic changes, respectively. Besides, the expression of NLRP3 inflammasome and IkB-*α*/NF-*κ*B pathway proteins was estimated by immunofluorescence, quantitative real-time PCR (qRT-PCR), and Western blotting assays, respectively. XFZYD pretreatment significantly ameliorated pulmonary ventilation function and reduced the CPB-induced lung histopathological injury, inflammatory cell infiltration in BALF and lung, and the apoptosis of lung cells. Interestingly, XFZYD decreased the CPB-induced NLRP3, ASC, Caspase-1 p20, Pro-GSDMD, GSDMD p30, IL-18, IL-1*β* p-P65, and p-IKB*α* mRNA or protein levels in lung tissues in ALI model rats. In summary, these findings suggest that XFZYD effectively mitigates NLRP3 inflammasome-dependent pyroptosis in CPB-induced ALI model rats, possibly by inhibiting the IkB-*α*/NF-*κ*B pathway in the lung.

## 1. Introduction

Cardiopulmonary bypass (CPB) is a necessary method for open-heart surgery. With the development of CPB technologies and surgical techniques, its safety has been greatly improved, and the incidence of mortality and complications has been gradually reduced. However, acute lung injury (ALI) after CPB is still an important complication, which is closely related to the success rate of cardiac surgery and postoperative mortality [1]. The clinical treatment of acute lung injury after CPB mainly includes controlled fluid therapy, protective ventilation strategies, and extracorporeal membrane oxygenation (ECMO). The supportive therapy is to reduce pulmonary edema and improve oxygenation with nitric oxide (NO) inhalation or pulmonary vascular reperfusion prostaglandin drugs and other antioxidants, which are anti-inflammatory. However, the existing measures can not reduce the incidence of ALI after CPB [2]. Therefore, the prevention and treatment of ALI after CPB are not only an urgent problem to be solved in the clinic, but also a hotspot of basic research.

It is currently believed that ischemia-reperfusion injury and systemic inflammatory response syndrome may be important causes of ALI after CPB [3, 4]. During the reperfusion period, diffuse neutrophil-dominated inflammatory cell infiltration and pulmonary edema rapidly in the lung tissues, and the symptoms of severe patients rapidly progressed to the impairment of alveolar epithelial-pulmonary vascular endothelial barrier function, which presents fatal pulmonary edema and severe pulmonary ventilation dysfunction. Almost all CPB patients had different degrees of acute lung damage and hypofunction after surgery, with mild cases having only transient symptoms, and severe cases presenting acute respiratory distress syndrome or even acute respiratory failure. When severe lung injury occurs, the fatality rate reaches more than 50% [5].

Alveolar epithelial cells and pulmonary microvascular endothelial cells, the key targets of ALI after CPB, are damaged by cell mitochondria, endoplasmic reticulum stress, and the accumulation of anaerobic hydrolysates during ischemia-reperfusion. Oxidative stress response in the early stage of reperfusion and reactive oxygen species (ROS) burst start innate immunity and activate aseptic inflammatory response. In the late stage of reperfusion injury, the initiation of innate immunity can progress within days to weeks, resulting in irreversible damage to organ function and structure [6, 7]. Numerous studies have shown that the root cause of ALI is acute inflammatory response syndrome (SIRS) caused by various factors [8]. Kinds of inflammatory cells are recruited and activated in the lung and release massive proinflammatory mediators, causing the cascade “waterfall effect” of inflammatory cytokines, forming a runaway inflammatory response. Therefore, the prevention and treatment strategy of ALI after CPB should mainly consider inhibiting the overexpression and overstrong effect of inflammatory factors [9, 10].

As the core part of the inflammatory response, inflammasome has attracted much attention. The inflammasome can be activated by different stimulation signals including pathogen-associated molecular patterns (PAMP) and danger-associated molecular patterns (DAMP), and activated inflammasomes can convert inactive procaspase-1 into active caspase-1 [11]. Caspase-1 can induce inflammatory cell death, namely, cell pyroptosis. In addition, pro-IL-1 and pro-IL-18 precursors can be cut to active IL-1 and IL-18, which promote the release of inflammatory factors, aggravate inflammatory response, and cause pathological inflammatory response [12]. The inflammasome consists of receptor protein, apoptosis-associated speck-like protein containing CARD (ASC), and procaspase-1. Receptor proteins of inflammasomes include NLRP1, NLRP3, and IPAF in the NOD-1ike receptors (NLRs) family, which is widely expressed in the cytoplasm, and absent in melanoma 2 (AIM2) in the HIN-200 family of cytoplasmic DNA sensors [13].

NLRP3 inflammasomes are the most widely studied inflammatory corpuscles at present. They are part of innate immunity. NLRP3 inflammasomes are located in cells and can feel harmful stimuli in the intracellular and extracellular environment. Toll-like receptors (TLRs) and C-type lectin receptors (CLRs) are the pattern recognition receptors (PPRs) on the surface of the cell membrane. NLRP3 inflammasome, TLRs, and CLRs jointly recognize harmful stimuli in the intracellular and extracellular environment, constitute the body's inherent immune defense system, which can identify a series of endogenous and exogenous stimulating factors, promote the occurrence of the inflammatory response, and aggravate tissue damage [14]. NLRP3 inflammasome plays important roles not only in the resistance of pathogenic microorganisms' invasion, but also in the progress of other diseases like tumor [15], coronary heart disease, and immune metabolic diseases [16, 17]. Therefore, the NLRP3 inflammasome is important in the pathogenesis of ALI. The correlation between NLRP3 inflammatory bodies and ALI after CPB has also become a research hotspot in the field of inflammation.

In recent years, the research on the prevention and treatment of ALI after CPB by Traditional Chinese medicine has gradually deepened, and the possible mechanism of traditional Chinese medicine in the treatment of ALI after CPB has been clarified through experiments, which proves the effectiveness of traditional Chinese medicine in this regard. According to the records of symptom description in traditional Chinese medicine literature of past dynasties and the understanding of modern medicine on its clinical manifestations and lesion evolution process, acute lung injury after CPB can be classified into the categories of “sudden asthma” and “asthma out” [18]. Combined with the theory of toxic evil blocking the lung and reversing the disorder of the Qi mechanism, it is concluded that early lung propaganda and detoxification are an important principle for the treatment of ALI after CPB, and the methods of clearing heat and detoxification and purging the bowels are often used [19]. Although Chinese medicine has made some progress in the prevention and treatment of ALI after CPB, it has not yet formed a complete theoretical system and mature methods. Therefore, it is necessary to continue the experimental and mechanism research on the prevention and treatment of ALI after CPB with traditional Chinese medicine, screen traditional Chinese medicine preparations with clear curative effects, and widely carry out clinical experiments and application research, to find more effective drugs for the prevention and treatment of CPB lung injury. XueFu ZhuYu Decoction (XFZYD) has the functions of promoting blood circulation and removing blood stasis, promoting qi, and relieving pain. It is composed of peach kernel, safflower, angelica, *Rehmannia glutinosa*, *Achyranthes bidentata*, *Ligusticum chuanxiong*, *Platycodon grandiflorum*, red peony, *Fructus aurantii*, *Bupleurum*, licorice, etc., which is a modified formula of Sini Powder and Taohong Siwu Decoction.

Previous studies have shown that XFZYD can significantly improve lung injury induced by sepsis and LPS [20]. XFZYD can reduce endothelial dysfunction and maintain endothelial function homeostasis by inhibiting ROS-induced oxidative stress, thereby inhibiting the inflammatory response and reducing apoptosis [21, 22]. Previous studies have reported that XFZYD can inhibit the activation of NLRP3 inflammatory bodies, inhibit the inflammatory response, and reduce ALI after CPB. Therefore, inhibiting the inflammatory response mediated by inflammatory corpuscles is an important mechanism of XFZYD against ALI after CPB, which suggests that inflammatory corpuscle pathway-mediated cell death is the potential mechanism of XFZYD against ALI after CPB. However, the regulatory effect and mechanism of XFZYD on inflammatory corpuscle activation in ALI after CPB need to be further clarified.

In this study, we proposed that the inflammasome-mediated cell pyroptosis pathway mediates ALI after CPB. On this basis, we clarify the activation of the classical focal death pathway in ALI after CPB at the cellular and overall level and further clarify its role in ALI after CPB, which provides a new idea for the intervention of ALI after CPB. It also provides experimental support for cell death to become an important target for the intervention of ALI after CPB. Based on the anti-inflammatory effect of XFZYD and its regulatory effect on the activation of inflammatory corpuscles in ALI after CPB, we propose the classical cell pyroptosis pathway, mediate the mechanism of XFZYD against ALI after CPB, and clarify the regulatory effect and mechanism of XFZYD on cell pyroptosis in ALI after CPB from the cellular and overall level to provide experimental support and scientific basis for the application and research of XFZYD.

## 2. Materials and Methods

### 2.1. XueFu ZhuYu Decoction Preparation

XueFu ZhuYu Decoction consists of peach kernel (12 g), safflower (9 g), angelica (9 g), *Rehmannia glutinosa* (9 g), *Achyranthes bidentata* (9 g), *Ligusticum chuanxiong* (4.5 g), *Platycodon grandiflorum* (4.5 g), red peony (6 g), *Fructus aurantii* (6 g), *Bupleurum* (3 g), and licorice (6 g). All of the drugs were provided by the Pharmacy of the third people's Hospital of Hangzhou (Zhejiang, China) and were fully confirmed by Pro. Xiao-Feng Yuan (Medical Research Institute of Traditional Chinese Medicine, Zhejiang Chinese Medical University, Zhejiang, China) according to the Chinese Pharmacopoeia 2015. Briefly, these materials were soaked in 1000 mL water for 30 minutes and then decocted with boiling water twice for 2 h each time. Finally, we mixed these two times filtered solutions together in a water-bath at 60°C and evaporated them to 100 mL. The concentration of the drug solution was 0.78 g crude drug/mL. The final extract was bottled and stored at 4°C for further use.

### 2.2. Animals and Treatment

A total of 30 healthy male Sprague Dawley rats (age: 7 weeks) weighing 180∼200 g were acquired from SLAC Laboratory Animal Co., Ltd. (license No.: SCXK (Hu) 2017–0005, Shanghai, China). Rats were housed in standard laboratory conditions and had free access to food and tap water. To establish the CPB model, we followed the previous study [4]. One week after adaptation, rats were randomly divided into the following groups (*n* = 6, in each group): sham, CPB, XFZYD, Ac-YVAD-CMK, and Bay-11-7082. Before CPB treatments, rats in sham and CPB groups were given by gavage once daily with the same amount of normal saline. The rats in the XFZYD (8.2 g/kg) group were intragastrically administered XFZYD once a day after XFZYD rewarming to 37°C. The dose of XFZYD for rats in XFZYD group was calculated with a body surface area normalization method in the light of normal clinical dose using the following formula: rats (g/kg) = (human dose (78 g crude drug/day)/human weight (60 kg)) × 6.3. The rats in the Ac-YVAD-CMK and Bay-11-7082 groups received a daily intraperitoneal injection of Ac-YVAD-CMK (2 mg/kg, Caspase 1 inhibitor; Sigma, USA) and Bay-11-7082 (5 mg/kg, NF-*κ*B inhibitor; Shanghai Rechem Science Co., Ltd. Shanghai, China). Each group was given intervention for 2 days. Thereafter, four time-points were set as follows: T0, before CPB; T1, the onset of the reopening of the left pulmonary artery; T2, after CPB treatment for 4 h; T3, after CPB treatment for 8 h. At each time-point, the blood samples (2 mL) were collected from the femoral artery, and then the arterial blood gas analysis was performed with GEM Premier 3000 Blood Gas Analyzer (Instrumentation Laboratory, MA) to calculate the oxygenation index (OI) and the respiratory index (RI). At T3, the rats were anesthetized with pentobarbital sodium (50 mg/kg, Sigma, USA), and the blood was obtained from the abdominal aorta and further centrifuged at 3000 rpm/min for 15 min to obtain serum. Finally, the right lung tissues were quickly removed and weighted for pathological, molecular, and biological studies.

### 2.3. ELISA

The levels of interleukin (IL)-1*β*, IL-6, IL-18, and tumor necrosis factor-*α* (TNF-*α*) in serum and supernatants of homogenate of the lung tissue were analyzed by commercial Enzyme-linked immunosorbent assay (ELISA) kits ThermoFisher Scientific, and Nanjing Jiancheng Bioengineering Institute, respectively.

### 2.4. Protein Levels and Neutrophil Count Analysis

After the rats were sacrificed, 1 ml of saline was placed into the right lung, and then a repeat suction was performed three times. The bronchoalveolar lavage fluid (BALF) was collected and centrifuged at 3000 rpm/min for 15 min at 4°C. Finally, the supernatant was collected, and then the protein levels in BALF were measured using a Bicinchoninic Acid (BCA) Protein Assay Kit (Beyotime, China). Cell sedimentation was resuspended in a 10 *μ*l PBS solution and the total cell number, and neutrophil counts were then performed in an automated cell differential count.

### 2.5. Lung Wet/Dry (W/D) Weight Ratio

The fresh left middle lobe was isolated, weighed, and then dehydrated for 48 h to record dry weight. The W/D weight ratio was used to assess the extent of pulmonary edema.

### 2.6. Histological Analysis

We dissected the upper lobe of the lung, fixed it in 4% paraformaldehyde (Sigma-Aldrich), and embedded it in paraffin, and 4 *μ*m slices were obtained, followed by hematoxylin/eosin staining. Subsequently, histopathological changes in the lung tissues were assessed with an optical microscope (×200). The histological indices were evaluated by two experienced pathologists using the 5-scale grading system: 0 = no lesion, 1 = slight damage, 2 = mild damage, 3 = moderate damage, and 4 = highly severe lesions [23].

### 2.7. Immunohistochemistry (IHC) and Immunofluorescence

After embedding the upper lobe of the lung tissues into paraffin, the 4 *μ*m sections were used for immunolabeling analysis. Briefly, the lung sections were permeabilized, blocked, and then incubated with a primary antibody against myeloperoxidase (MPO, 1 : 1000, ab208670, Abcam, USA) overnight at 4°C, followed by incubation with a corresponding secondary antibody at ordinary temperature for 1 h. Images were obtained using an optical microscope at ×200 magnification. For immunofluorescence labeling, lung sections were treated with a primary antibody against F4/80 (1 : 1000, ab6640, Abcam), NLRP3 (1 : 500, DF7438, Affinity), ASC (1 : 500, sc-514414, Santa Cruz Biotechnology, INC), and Caspase 1 (1 : 500, DF6148, Affinity) and then with an ALexa Fluor 488-conjugated secondary antibody. The nuclei were stained for DAPI for 10 min in dark, and the detection of F4/80, NLRP3, ASC, and Caspase 1 proteins was examined using an IX71 fluorescence microscope (Olympus, Japan). The relative fluorescence intensity of positive cells was quantified using Image-Pro Plus software.

### 2.8. Terminal Deoxynucleotidyl Transferase dUTP Nick End Labeling (TUNEL) Analysis

Based on the manufacturer's operating steps, the apoptosis of the upper lobe of the lung tissues was detected using a TUNEL fluorescence FITC kit (Roche, USA). After TUNEL staining, the nuclear was stained using DAPI (1 : 100, Beyotime, China). Finally, the stained sections are photographed by fluorescence microscope and counted apoptotic cells.

### 2.9. qRT-PCR Analysis

Total RNA was extracted from the inferior lobe of the lung tissues by using TRIzol reagent (Invitrogen, USA). After RNA transcribed, qRT-PCR was performed using SYBR Premix Ex Taq II (Takara) on a Roche Light Cycler 480 system (Roche, Bedford, MA, USA). The primers used were as follows: NLRP3: forward 5-GAACCTCGAGCTGATCTTCG-3, reverse 5-CTGAGCT CCGATTCGAAGG-3; ASC: forward 5-GCCCACCAACC CAAGCAAGATG-3, reverse 5-CTCCGCTCCAGGTCC TCCAC-3; GSDMD: forward 5-GCCTCCACAACTTCCT GACAGATG-3, reverse 5-GGTCTCCACCTCTGCCCGT AG-3; Caspase 1: forward 5-GAACCTCGAGCTGAT CTTCG-3, reverse 5-CTGAGCTCCGATTCGAAGG-3; *β*-actin: forward 5-TCAGGTCATCACTATCGGCAAT-3, reverse 5-AAAGAAAGGGTGTAAAACGCA-3. *β*-actin was used as an internal control, and relative levels of targets were measured using the comparative 2^ − ΔΔCt^ method.

### 2.10. Western Blot Analysis

The inferior lobe of the lung tissues was homogenized in RIPA lysis buffer (Thermo Fisher Scientific, Inc.). After that, 30 *μ*g protein was separated in 10% SDS-PAGE. Proteins were then transferred to PVDF membranes (Millipore). Then, the PVDF membranes were blocked with 5% non-fat dry milk for 1 h at room temperature and then incubated with primary antibodies against p-P65 (1 : 1000, AF2006, Affinity); P65 (1 : 1000, AF5006, Affinity); p-IKB*α* (1 : 1000, AF2002, Affinity); IKB*α* (1 : 1000, AF5002, Affinity); NLRP3 (1 : 1000, DF7438, Affinity), ASC (1 : 2000, sc-514414, Santa Cruz Biotechnology, INC), Caspase-1 p20 (1 : 2000, AF4005, Affinity), Pro-GSDMD (1 : 1000, AF4012, Affinity), GSDMD p30 (1 : 1000, DF12275, Affinity), IL-18 (1 : 1000, DF6252, Affinity), IL-1*β* (1 : 1000, AF5103, Affinity), and *β*-actin (1 : 5000, AF7018, Affinity) at 4°C overnight. Then, the membranes were then incubated with the appropriate secondary antibodies at room temperature for 2 h. *β*-actin was selected as the loading control. Finally, the protein bands were caught by using a Chemiluminescence image analysis system (Tanon, Shanghai, China) and analyzed with the Image J software (National Institutes of Health, USA).

### 2.11. Statistical Analysis

Data were presented as mean ± standard deviation (SD). Differences between the groups were analyzed for statistical significance using the one-way analysis of variance (ANOVA) followed by the Least Significant Difference (LSD) test using SPSS 22.0 software (SPSS, USA). *P* < 0.05 was considered statistically significant.

## 3. Results

### 3.1. Effect of XFZYD on Blood Gas Analysis in CPB-Induced ALI Rats

**Figure 1:**
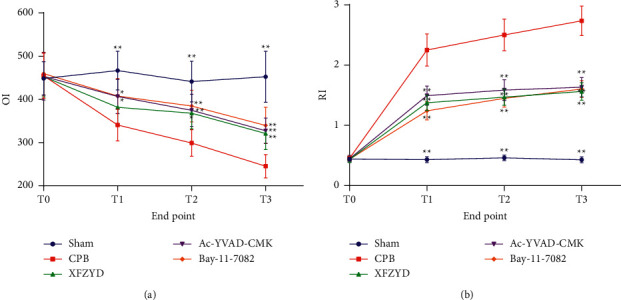
XFZYD improved lung function in rat models with CPB-induced ALI. (a) Comparisons of oxygenation index (OI) among groups at different time-points. (b) Comparisons of the respiratory index (RI) among groups at different time points. Data are presented as the mean ± SD. *N* = 6/group. ^*∗*^*P* < 0.05; ^*∗∗*^*P* < 0.01, vs. CPB group.

To detect the effects of XFZYD on pulmonary function, we examined the oxygenation index and respiratory index in XFZYD-pretreated rats after treatment with CPB. The blood gas analysis results showed that OI at T1, T2, and T3 points was significantly decreased (Figure 1(a)), and RI was significantly increased in model rats (Figure 1(b)). Compared with the time-point of T1, T2, and T3 in the CPB group, OI of XFZYD, Ac-YVAD-CMK, and Bay-11-7082 groups was significantly increased, while RI was the opposite. Interestingly, there was no significant difference in OI and RI between the XFZYD, Ac-YVAD-CMK, and Bay-11-7082 groups. The above-mentioned results suggested that XFZYD might possess a protective effect on pulmonary function in CPB-induced ALI rats by inhibiting inflammasome activation and subsequent pyroptosis.

### 3.2. Effect of XFZYD on Inflammatory Reaction in Serum and Lung Tissue of ALI Rats

**Figure 2:**
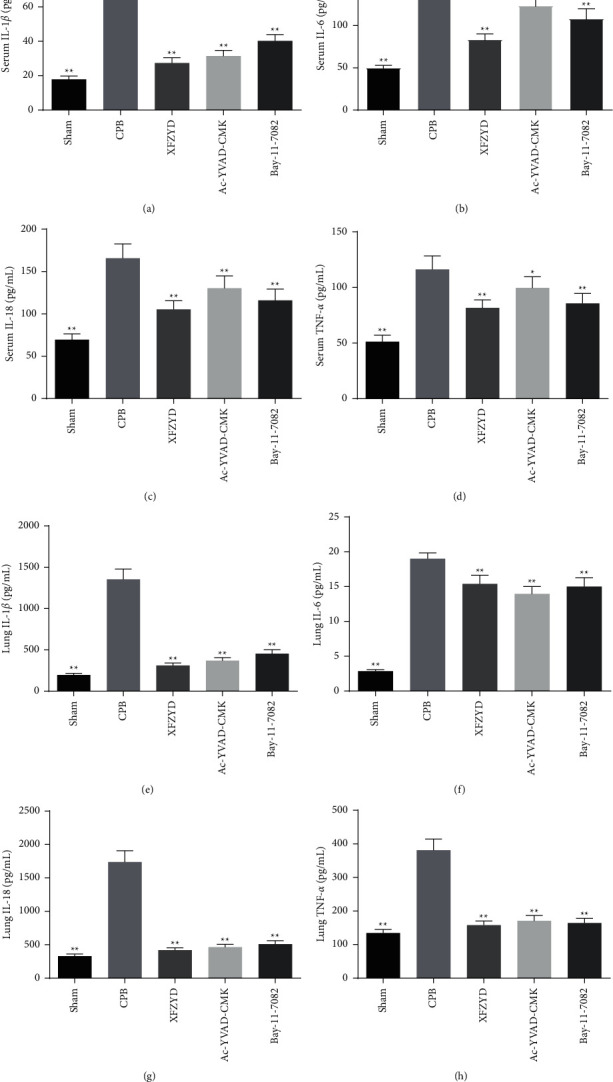
XFZYD decreased inflammatory cytokine levels in rat models with CPB-induced ALI. IL-1*β*, IL-6, IL-18, and TNF-*α* levels in serum (a–d) and lung tissues (e–h) were detected by ELISA assays, respectively. Data are presented as the mean ± SD. *N* = 6/group. ^*∗*^*P* < 0.05; ^*∗∗*^*P* < 0.01, vs. CPB group.

**Figure 3:**
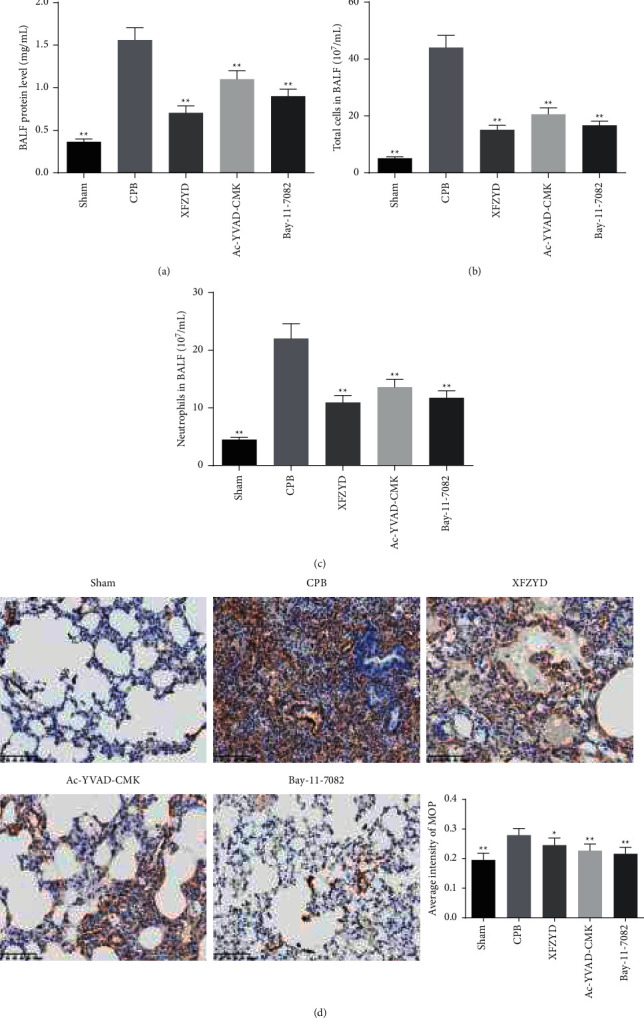
XFZYD attenuated pulmonary vascular leakage and inflammatory cell infiltration levels in rat models with CPB-induced ALI. (a) Protein, (b) the number of total cells, and (c) neutrophils in bronchoalveolar lavage fluid (BALF) at 8 h after the CPB operation were measured to estimate pulmonary vascular leakage. (d) Immunohistochemistry was employed to estimate the average intensity of myeloperoxidase (MPO) in lung tissues of rats with CPB-induced acute lung injury. Scare bar = 100 *μ*m. The data are representative images and expressed as mean ± SD of each group of rats (*n* = 6 per group). ^*∗*^*P* < 0.05; ^*∗∗*^*P* < 0.01, vs. CPB group.

Compared with the sham group, the levels of IL-1*β*, IL-6, IL-18, and TNF-*α* were distinctly increased in serum and lung tissue of CPB-induced ALI rats. Furthermore, XFZYD, Ac-YVAD-CMK, and Bay-11-7082 treatment remarkably suppressed the release of inflammatory cytokines, as shown in Figures 2(a)–2(h). Additionally, BALF was collected to analyze the extent of lung inflammation. As depicted in Figures 3(a)–3(c), the BALF protein levels, the number of total cells, and neutrophils in BALF were significantly increased in the CPB group compared with the sham group. Meanwhile, pretreatment with XFZYD, Ac-YVAD-CMK, and Bay-11-7082 in CPB-challenged rats significantly decreased the BALF protein levels, the number of total cells and neutrophils in BALF. Also, lung MPO activity is an indicator of neutrophil infiltration. Then, the MPO activity was evaluated in the lung tissue using immunohistochemical staining. Consistent with the number of neutrophils in BALF, we found that the protein levels of MPO were significantly increased in the CPB group. However, compared to the CPB group, XFZYD, Ac-YVAD-CMK, and Bay-11-7082 administration significantly decreased the MPO activity in lung tissue (Figure 3(d)).

### 3.3. Effect of XFZYD on the Histological Injury of Lung Tissue in CPB-Induced ALI Rats

**Figure 4:**
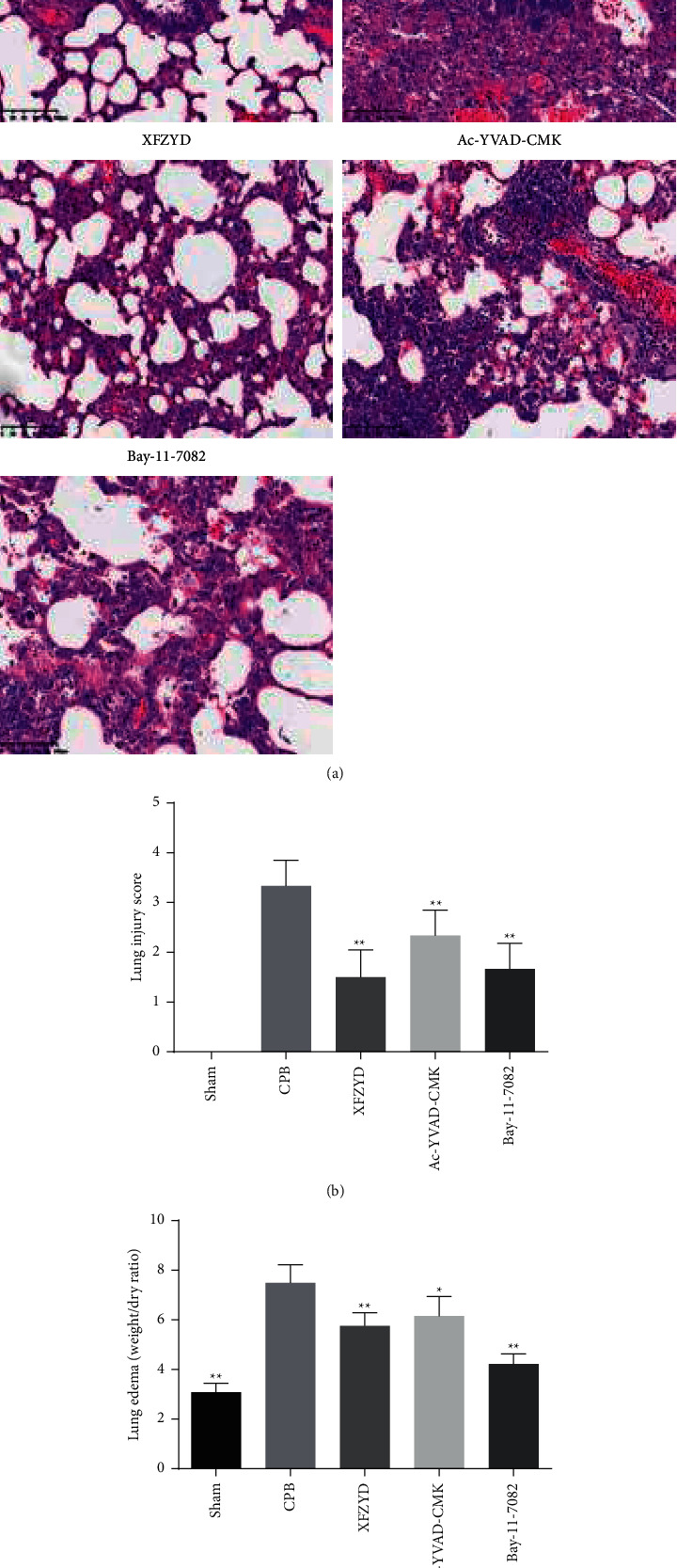
XFZYD ameliorated pathological changes of lung tissues in rat models with CPB-induced ALI. (a) Representative images of lung sections with hematoxylin and eosin staining (scare bar, 100 *μ*m). (b) Lung injury scores were quantified and shown in the different groups. (c) Lung wet/dry weight ratio. Data are presented as mean ± SD. ^*∗*^*P* < 0.05; ^*∗∗*^*P* < 0.01, vs. CPB group.

At 8 h after CPB, the typical histological injury was observed under a light microscope as shown in Figure 4(a). The alveolar structure of the sham group was clear and complete. In the CPB group, the histological changes of the lungs included severe alveolar and mesenchymal edema, alveolar cavity collapse, abnormalities in the infiltration of inflammatory cells, and erythrocyte exudation. Compared with the CPB group, the pretreatment with XFZYD, Ac-YVAD-CMK, and Bay-11-7082 significantly reduced the pathological damage, as shown by a significant decrease in cellular infiltration, lessened alveolar injury, and red blood cell exudation. Moreover, as shown in Figure 4(b), lung injury scores in the CPB group were significantly increased compared with the sham group. Compared with the CPB group, the lung injury score of the XFZYD, Ac-YVAD-CMK, and Bay-11-7082 groups was significantly reduced, particularly in the XFZYD group. Similarly, the tissue wet/dry weight ratio in lung tissues was worsened in the CPB group, but in XFZYD, Ac-YVAD-CMK, and Bay-11-7082 groups, it was lower than that in the CPB group (Figure 4(c)).

### 3.4. Effect of XFZYD on the Cell Apoptosis and Macrophage Infiltration of Lung Tissue in CPB-Induced ALI Rats

**Figure 5:**
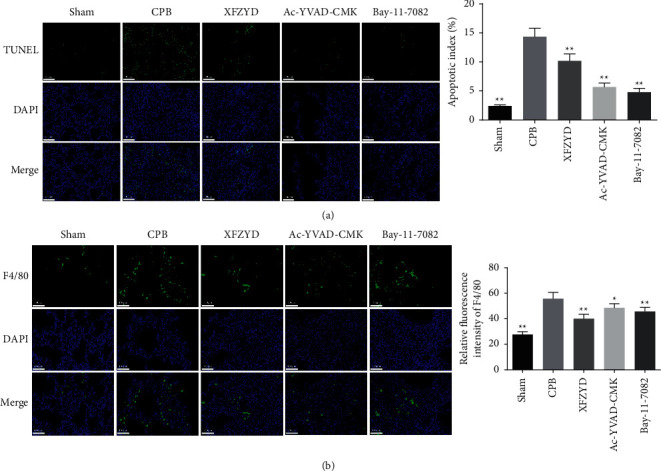
XFZYD inhibited cell apoptosis and macrophage infiltration in rat models with CPB-induced ALI. (a) Representative images of TUNEL stain and quantitative results. (b) Representative immunofluorescence images show the expression levels of F4/80 in lung tissues of rats with CPB-induced acute lung injury. Values are expressed as mean ± SD. ^*∗*^*P* < 0.05; ^*∗∗*^*P* < 0.01, vs. CPB group.

The apoptosis and macrophage infiltration of lung tissue were detected by TUNEL staining and F4/80 immunofluorescence staining, respectively. As shown in Figure 5(a), substantial apoptotic cells were found in the CPB group, and the apoptotic index was significantly higher than that in the sham group. As expected, the apoptotic index of the XFZYD, Ac-YVAD-CMK, and Bay-11-7082 groups was observably lower than that of the CPB group, especially the Bay-11-7082 group. Consistent with a similar result of MPO activity, the results of immunofluorescence showed a large number of F4/80-positive cells in CPB-induced injured lungs, which was significantly reduced by XFZYD, Ac-YVAD-CMK, and Bay-11-7082 treatment, respectively (Figure 5(b)). These results indicate that XFZYD attenuated inflammatory response and cell apoptosis in lung tissue of CPB-induced ALI rats.

### 3.5. Effect of XFZYD on the NLRP3 Inflammasome Activation and IKB*α*/NF-*κ*B Signaling Pathway of Lung Tissue in CPB-Induced ALI Rats

**Figure 6:**
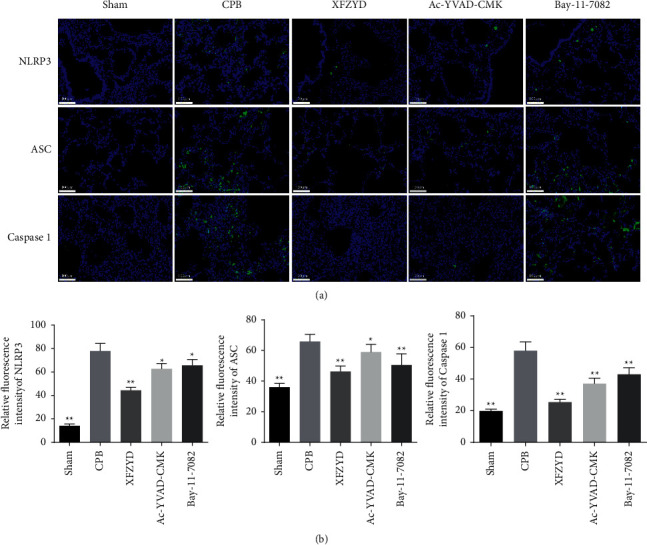
XFZYD decreased pyroptosis in rat models with CPB-induced ALI. (a) Pyroptosis-related proteins in the lung tissues of rats with CPB-induced acute lung injury, including NLRP3, ASC, and caspase 1 were examined by immunofluorescence analysis. (b) Quantitative analysis of NLRP3, ASC, and caspase 1 expression. Scare bar = 100 *μ*m. Values are expressed as mean ± SD. ^*∗*^*P* < 0.05; ^*∗∗*^*P* < 0.01, vs. CPB group.

**Figure 7:**
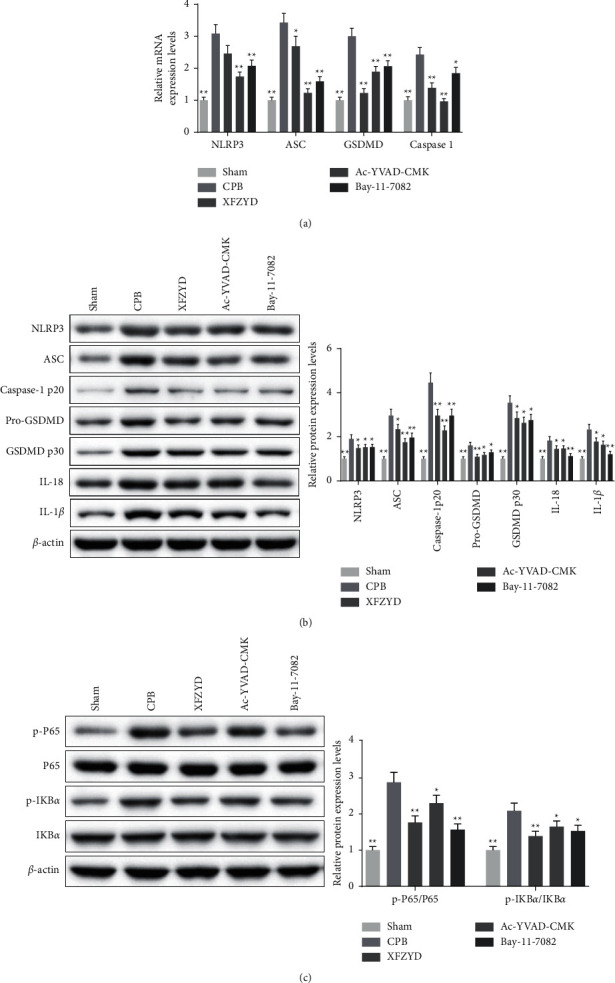
XFZYD inhibited the IKB*α*/NF-*κ*B signaling in rat models with CPB-induced ALI. (a) The mRNA expression levels of NLRP3, ASC, GSDMD, and caspase 1 in CPB-induced lung injury rats treated with XFZYD, Ac-YVAD-CMK, and Bay-11-7082 were measured via RT-qPCR. (b and c) western blots were performed to determine NLRP3, ASC, Caspase-1 p20, Pro-GSDMD, GSDMD p30, IL-18, IL-1*β* p-P65, P65, p-IKB*α*, and IKB*α* levels in lung tissues of rats with CPB-induced acute lung injury. *β*-actin was used as a loading control for the blots. All the data were presented as the means ± SD. from independent experiments performed in triplicate. ^*∗*^*P* < 0.05; ^*∗∗*^*P* < 0.01, vs. CPB group.

To further determine the mechanism of the therapeutic effect of XFZYD on CPB- associated lung injury, the NLRP3 inflammasome activation- and IKB*α*/NF-*κ*B signaling pathway-related targets were assessed by immunofluorescence staining, qPCR assays, and Western blotting. We found that the relative fluorescence intensity of NLRP3, ASC, and Caspase 1 (Figures 6(a) and 6(b)), as well as the mRNA expression of NLRP3, ASC, GSDMD, and Caspase 1, significantly increased in the lungs of CPB- treated rats but was counteracted by XFZYD, Ac-YVAD-CMK, and Bay-11-7082 pretreatment (Figure 7(a)). The release of large quantities of IL-18 and IL-1*β* requires the GSDMD p30 (the cleaved form of GSDMD), which is the “executioner” of pyroptosis [24]. Consistent with the immunofluorescence staining and qPCR assay results, the western blotting assay showed that the expression of NLRP3, ASC, Caspase-1 p20, Pro-GSDMD, GSDMD p30, IL-18, and IL-1*β* protein was markedly increased in the CPB group. When compared with the CPB group, NLRP3, ASC, Caspase-1 p20, Pro-GSDMD, GSDMD p30, IL-18, IL-1*β* expressions were evidently decreased in XFZYD, Ac-YVAD-CMK, and Bay-11-7082 groups (Figure 7(b)). These results indicate that the NLRP3 inflammasome was activated in CPB-induced ALI, which was attenuated by XFZYD, Ac-YVAD-CMK, and Bay-11-7082 pretreatment. In addition, the inhibitor of the I*κ*B*α*/NF‐*κ*B signaling pathway is widely accepted to play important role in inflammation [25], and it is also associated with the progression of ALI [26]. In this study, the proteins related to IKB*α*/NF-*κ*B signaling pathway were assessed by western blot assay. Results of western blot showed that the expressions of p-IKB*α* and p-P65 in lung tissue of CPB-induced ALI rats were significantly upregulated, while they were downregulated after XFZYD, Ac-YVAD-CMK, and Bay-11-7082 intervention (Figure 7(c)). The results showed that they attenuated CPB-induced NLRP3 inflammasome activation by inhibiting IKB*α*/NF-*κ*B signaling pathway in ALI.

## 4. Discussion

In this study, we investigated the effects and potential mechanism of XFZYD in the pathogenesis of ALI after CPB. Also, this study confirmed the potential effect of XFZYD against CPB-induced ALI and explored its underlying mechanism that is involved with NLRP3 inflammasome-dependent pyroptosis by targeting the IkB-*α*/NF-*κ*B pathway. It has been reported that CPB is often associated with a systemic inflammatory response syndrome, significantly affecting pulmonary function [27]. Also, Chen et al. reported that triptolide, an active natural product derived from *Tripterygium wilfordii,* improved neurobehavioral functions, neuroinflammation, and oxidative stress via activating Nrf2 pathway and inhibited NF-*κ*B pathway in a rat model of cardiopulmonary bypass with deep hypothermia circulatory arrest [28]. OI and RI are widely used to assess lung function in clinical applications, with RI being negatively correlated with OI. In the current study, compared with sham group, in CPB group, the OI was substantially downregulated, the RI was sensibly upregulated, and the lung wet to dry mass ratio was observably increased after 8 h of CPB. These results suggested that there are lung ventilation function abnormality and pulmonary interstitial edema in CPB-induced ALI rats. Compared with CPB group, the above-mentioned indicators in XFZYD, Ac-YVAD-CMK, and Bay-11-7082 groups were significantly improved. Additionally, it is noteworthy that there was no significant difference in OI and RI among the XFZYD, Ac-YVAD-CMK, and Bay-11-7082 groups. It indicates that the XFZYD can enhance lung ventilation function and attenuate the pulmonary interstitial edema through inhibiting inflammatory response activation in CPB-induced ALI rats.

More importantly, the present study demonstrates that XFZYD pretreatment attenuates the production of IL-1*β*, IL-6, IL-18, and TNF-*α* in the serum and lung tissues of CPB-induced ALI rats. A study by Kasper et al. demonstrated that the antioxidant and anti-inflammatory green tea polyphenol (-)-epigallocatechin-3-gallate significantly attenuated lung edema and pulmonary neutrophil infiltration in CPB piglets [29]. Similarly, our results revealed that CPB-induced ALI increased the protein and the number of inflammatory cells in BALF of rats, which were significantly inhibited by XFZYD treatment. Moreover, the neutrophils and macrophage infiltration into lung tissues play an important role in the inflammatory process in ALI [30, 31]. Besides, MPO activity is a commonly used index to estimate the degree of neutrophils infiltration into tissues [24, 32]. Our data demonstrated that XFZYD significantly lowered the abundance of neutrophils and macrophages. Furthermore, our results also suggest that the architecture of lung tissues in CPB group had changed with interstitial edema, alveolar injury, red blood cell exudation, and neutrophil infiltration, whereas the results indicated that XFZYD pretreatment prevented the morphological changes of lung injury and suppressed the neutrophil infiltration in lung tissues of CPB rats. Taken together, these results demonstrated that XFZYD pretreatment attenuated pulmonary injury in CPB-induced ALI rats, which indicated that the use of XFZYD may be an alternative therapeutic avenue for patients with ALI during CPB.

Recent findings have demonstrated that apoptosis, a form of programmed cell death, plays an important role in CPB [33, 34]. Xu et al. showed that pretreatment of Xuebijing injection further reduced the apoptosis of type II alveolar epithelial cells after CPB [35]. In this study, TUNEL staining for apoptotic cells indicated that there was a striking increase in the lung tissues after CPB, indicating that cell death after CPB was related to the process of lung injury. Meanwhile, the apoptotic index in Ac-YVAD-CMK group, a specific caspase-1 inhibitor, was significantly lower than that in XFZYD group, further suggesting that inhibiting apoptosis and pyroptosis may play a protective role in ALI. Pyroptosis is associated with the caspase-1 and the release of IL-1*β* and IL-18 [36]. It is noteworthy that pyroptosis is related to diverse ischemia-reperfusion injury-associated diseases, including CPB-induced ALI [37–39]. As known, the NLRP3 inflammasome, a protein complex consisting of NLRP3, caspase-1, and ASC, could sense intracellular and extracellular dangerous signals [40]. Additionally, activated caspase-1 also cleaves gasdermin D (GSDMD), which leads to the perforation of cells and pyroptosis [41]. Inhibiting the molecular events of the NLRP3 inflammasome is identified as the potential treatment strategy for ALI. Liang et al. reported that lycorine ameliorated bleomycin-induced pulmonary fibrosis by inhibiting NLRP3 inflammasome activation and pyroptosis through targeting the PYD domain of ASC [42]. Yang et al. found that corticosteroids inhibited the alveolar structure destruction, infiltration of neutrophils, and the inflammatory cytokines release of IL-1*β* and IL-18 in lung by suppressing both NF-*κ*B signal pathway and mtROS-dependent NLRP3 inflammasome activation in LPS-induced murine ALI model [43]. In this study, we found that the lung tissue NLRP3, ASC, GSDMD, Caspase 1 mRNA levels, and NLRP3, ASC, Caspase 1 p20, Pro-GSDMD, GSDMD p30, IL-18, and IL-1*β* protein levels in CPB group were significantly higher than those in sham group. Interestingly, each index in XFZYD, Ac-YVAD-CMK, and Bay-11-7082 groups was significantly decreased when compared with CPB group, especially in Ac-YVAD-CMK, and Bay-11-7082 groups. These findings suggest that the alleviation of CPB-induced ALI by XFZYD pretreatment may be related to its inhibition of cell pyroptosis-mediated inflammatory response in lung tissue.

NF-*κ*B, a key regulator of cellular inflammation balance, has emerged as an important therapeutic target for various diseases, and the inhibition of NF-*κ*B can alleviate the ALI [44, 45]. Inhibitory proteins of the I*κ*B play an important role in the homeostasis of NF-*κ*B [46]. Furthermore, NF-*κ*B activation induced by phosphorylated I*κ*B contributes to the imbalance of transcription genes to activate NLRP3 and thus promotes the release of inflammatory factors [46, 47]. Accumulating evidence has found that the activation of NF-*κ*B p65 was associated with the pathological process of ALI. For example, Zhao et al. revealed that NF-*κ*B might raise levels of miR-99b and contribute to ALI in LPS-induced mice [48]. Also, Chen et al. reported that LPS enhanced phosphorylated (p)-I*κ*B and p-p65 levels in NR8383 cells and lung tissues [49]. Hirao et al. clarified that recombinant human soluble thrombomodulin significantly decreased NF-*κ*B p65 expressions in lung tissue of CPB rats [50]. Similarly, the present results show that XFZYD could antagonize the increase of p-p65 and p-IKB*α* protein expression induced in the lung of CPB-induced ALI rats. Generally, these results suggest that XFZYD may inhibit CPB-induced inflammatory response and NLRP3-related pyroptosis through the inactivation of the IKB*α*/NF-*κ*B pathway.

## 5. Conclusion

In summary, the current study demonstrates that XFZYD pretreatment, in a way, ameliorates the acute lung injury in rats after CPB, owing to the ability of XFZYD in inhibiting inflammatory reactions, apoptosis, and NLRP3 inflammasome-dependent pyroptosis of lung tissues, likely through inactivating the IKB*α*/NF-*κ*B pathway. Our results illustrated that XFZYD may be a potential novel treatment for the prevention of CPB-induced ALI. However, this study has several limitations. Further research is essential for determining the influences of XFZYD.

## Data Availability

The data analyzed during the present study are available from the corresponding author upon reasonable request.
